# Elimination of Cancer Stem-Like “Side Population” Cells in Hepatoma Cell Lines by Chinese Herbal Mixture “Tien-Hsien Liquid”

**DOI:** 10.1155/2012/617085

**Published:** 2012-10-14

**Authors:** Chih-Jung Yao, Chi-Tai Yeh, Liang-Ming Lee, Shuang-En Chuang, Chuan-Feng Yeh, Wan-Ju Chao, Tung-Yuan Lai, Gi-Ming Lai

**Affiliations:** ^1^Cancer Center, Wan Fang Hospital, Taipei Medical University, Taipei 11696, Taiwan; ^2^Center of Excellence for Cancer Research, Taipei Medical University, Taipei, Taiwan; ^3^Department of Internal Medicine, School of Medicine, College of Medicine, Taipei Medical University, Taipei 11031, Taiwan; ^4^Department of Surgery, Shuang Ho Hospital, Taipei Medical University, Taipei, Taiwan; ^5^Graduate Institute of Clinical Medicine, Taipei Medical University, Taipei, Taiwan; ^6^Graduate Institute of Medical Sciences, National Defense Medical Center, Taipei, Taiwan; ^7^National Institute of Cancer Research, National Health Research Institutes, Miaoli, Taiwan; ^8^Graduate Institute of Pharmacognosy, College of Pharmacy, Taipei Medical University, Taipei, Taiwan; ^9^Department of Traditional Medicine, Wan Fang Hospital, Taipei Medical University, Taipei, Taiwan

## Abstract

There are increasing pieces of evidence suggesting that the recurrence of cancer may result from a small subpopulation of cancer stem cells, which are resistant to the conventional chemotherapy and radiotherapy. We investigated the effects of Chinese herbal mixture *Tien-Hsien Liquid* (THL) on the cancer stem-like side population (SP) cells isolated from human hepatoma cells. After sorting and subsequent culture, the SP cells from Huh7 hepatoma cells appear to have higher clonogenicity and mRNA expressions of stemness genes such as *SMO, ABCG2, CD133*, **β**-*catenin,* and *Oct-4* than those of non-SP cells. At dose of 2 mg/mL, THL reduced the proportion of SP cells in HepG2, Hep3B, and Huh7 cells from 1.33% to 0.49%, 1.55% to 0.43%, and 1.69% to 0.27%, respectively. The viability and colony formation of Huh7 SP cells were effectively suppressed by THL dose-dependently, accompanied with the inhibition of stemness genes, e.g., *ABCG2, CD133,* and *SMO*. The tumorigenicity of THL-treated Huh7 SP cells in NOD/SCID mice was also diminished. Moreover, combination with THL could synergize the effect of doxorubicin against Huh7 SP cells. Our data indicate that THL may act as a cancer stem cell targeting therapeutics and be regarded as complementary and integrative medicine in the treatment of hepatoma.

## 1. Introduction

In recent years, there has been increasing evidence supporting the notion that tumors are organized in hierarchical heterogeneous cell populations [1]. The capability to sustain tumor growth resides exclusively in a small proportion of tumor cells termed cancer stem cells (CSCs) or tumor-initiating cells [2], which have the properties of self-renewal, multilineage differentiation capacity, and, most importantly, the tumorigenicity. More importantly, recent researches show that CSCs are much more resistant to conventional cancer therapy than the other bulk cancer cells. CSCs have been considered to be the main cause for the failure of cancer treatment. Searching for the CSCs targeting therapeutics becomes a new strategy and challenge for improving the clinical outcome of cancer patients.

One common method to isolate the CSCs for therapeutics screening is the side population (SP) technique, which is originally used to detect the hematopoietic stem cells in bone marrow. This technique detects the so-called SP cells by dual-wavelength flow cytometry on the basis of the ability of these cells to efflux the fluorescent DNA-binding dye Hoechst 33342 [3]. The SP phenotype is characterized by breast-cancer-resistant protein-1 (BCRP1 or ABCG2), one of ATP-binding cassette (ABC) transporters, which is associated with multidrug resistance in many cancers by pumping out the drugs [4]. Since multidrug resistance is an *important characteristic of* CSCs, it has also been shown that the SP from cancer cells is enriched by CSCs [5]. Thus, SP cells are postulated to be a source of CSCs and represent an important potential target for cancer therapy. Recent work has led to the detection of the SP in a variety of tumor types, including leukemia, glioma, medulloblastoma, hepatoma, as well as breast, prostate, thyroid, colorectal, and ovarian carcinoma [6].

Lots of phytochemicals from fruits, vegetables, and herbs have anticancer activities and represent a promising therapeutic approach for the prevention and treatment of many cancers. The effects of phytochemicals on inhibiting tumor formation are well demonstrated both *in vitro* and *in vivo* [7]. Many of these compounds, such as berberine, curcumin, piperine, and cannabinoids [8–10], had been reported to eliminate cancer-stem-like cells. Natural products like herbal medicines, which possess evidence of molecular anticancer effects, may be considered as a potential source of therapeutics targeting on CSCs.

The *Tien-Hsien Liquid* (THL) is a Chinese herbal mixture, which has been used as a complementary anticancer agent for more than 10 years worldwide. It is aqueous preparation of herbal mixture and consists mainly of extracts from 14 Chinese herbs: *Cordyceps sinensis* (CS), *Oldenlandia diffusa* (OD), *Indigo pulverata levis* (IPL), *Polyporus umbellatus* (PU), *Radix astragali* (RA), *Panax ginseng* (PG), *Solanum nigrum L.* (SNL), *Pogostemon cablin* (PC), *Atractylodis macrocephalae rhizoma* (AMR), *Trichosanthes radix* (TR), *Clematis radix* (CR), *Margarite* (M), *Ligustrum lucidum Ait* (LLA), and *Glycyrrhiza radix* (GR). The biological activities of these herbs have been reported individually, including antioxidation, immunomodulation, antimutagenesis as well as cytostatic or cytotoxic effects. Recently, THL had been shown to induce apoptosis in many types of cancer cells and activate caspase-8, -9, and -3 in H1299 lung cancer cells [11]. Its effects on targeting PML-RAR*α* and oncogenic signaling pathways in acute promyelocytic leukemia NB4 cells had been demonstrated in our previous study [12]. More recently, its inhibitory effects on the metastasis, angiogenesis, and tumor growth had been reported by Chia et al. [13]. Regarding the crucial role of CSCs in the metastasis and progression of tumors [14], it is worthy and interesting to explore the effects of THL on the elimination of CSCs.

In this study, we separated and characterized cancer stem-like SP cells from human hepatoma cell lines to investigate the effects of THL on CSCs elimination. Our data indicate that THL could eliminate the cancer stem-like SP cells, accompanied with the suppressions of stemness genes expression, colony formation as well as tumorigenicity. These results further elucidate the mechanisms underlying the anticancer effects of this Chinese herbal mixture, which suggests its potential role as complementary medicine for cancer treatment.

## 2. Materials and Methods

### 2.1. Preparation of THL

THL was provided by Feida Union Pharmaceutical Manufactory, El Monte, CA. It is an aqueous preparation of herbal mixture and consists mainly of extracts from 14 Chinese medicinal herbs as mentioned previously. The original THL aqueous solution was lyophilized, weighed, and then stored in −20°C. It was reconstituted with sterile distilled water to prepare the working solutions and added to the appropriate medium to the final concentrations of 0.05, 0.25, 0.5, 2 mg/mL for the treatment of cultured cancer cells.

### 2.2. Culture of Hepatoma Cell Lines

The human hepatoma cell lines were obtained from the Bioresource Collection and Research Center (BCRC, Food Industry Research and Development Institute, Hsinchu, Taiwan). The cells were cultured in Dulbeco's modified Eagle's medium (DMEM) (Invitrogen Life Technologies, Carlsbad, CA) containing 10% fetal bovine serum (FBS) and 1% penicillin/streptomycin (Invitrogen) and incubated at 37°C in an atmosphere containing 5% CO_2_.

### 2.3. Side Population Analysis and Purification Using Flow Cytometry

The hepatoma cells were detached from the dishes with Trypsin-EDTA (Invitrogen) and suspended at 1 × 10^6^ cells/mL in Hank's balanced salt solution (HBSS) supplemented with 3% fetal calf serum and 10 mM HEPES. These cells were then incubated at 37°C for 90 minutes with 20 *μ*g/mL Hoechst 33342 (Sigma Chemical, St. Louis, MO), either alone or in the presence of 50 *μ*M verapamil (Sigma), which is an inhibitor of verapamil-sensitive ABC transporter. After 90-minute incubation, the cells were centrifuged immediately for 5 minutes at 300×g, 4°C and resuspended in ice-cold HBSS. The cells were kept on the ice to inhibit efflux of Hoechst dye and 1 *μ*g/mL propidium iodide (BD Pharmingen, San Diego, CA) was then added to discriminate dead cells. Finally, these cells were filtered through a 40 *μ*m cell strainer (BD Falcon) to obtain single-suspension cells. Cell dual-wavelength analysis and purification were performed on a dual-laser FACS Vantage SE (BD). The Hoechst 33342 was excited by 355 nm UV light and collect blue fluorescence with a 450/20 band-pass filter and red fluorescence with a 675 nm edge filter long pass (EFLP). A 610 nm dichroic mirror short pass (DMSP) was used to separate the emission wavelengths. The propidium iodide-positive dead cells were excluded from the analysis.

### 2.4. Culture of SP Cells into Tumor Spheres

After sorting, Huh7 side population cells were seeded with a density of 500 cells/well in 6-well ultra low attachment plates (Corning Inc., Corning, NY) in DMEM/F12 medium supplemented with B27 supplement (Invitrogen, Carlsbad, CA), bFGF (20 ng/mL, Invitrogen, Carlsbad, CA), and EGF (20 ng/mL, Millipore, Bedford, MA). After culture for 14 days, spheres were quantitated by inverted phase contrast microscopy.

### 2.5. Colony Formation of SP and Non-SP Cells

Freshly sorted SP and non-SP cells were counted, plated in triplicate at 200 cells per well in 6-well plates, and cultured in the medium described in Section 2.4 for 14 days. After most colonies had expanded to >50 cells, they were washed twice with PBS, fixed in methanol for 15 min, and dyed with crystal violet for 15 min at room temperature to visualize colonies for counting. Colony number and size were scored with the ChemiDoc-XRS imager, using the QuantityOne software package (BioRad, Hercules, CA). The declined colony counts represented the inhibitory effects of THL on colony formation of Huh7 SP cells.

### 2.6. Determining the Cell Viability by Sulforhodamine B (SRB) Assay

Both the SP and non-SP cells were seeded in 96-well plate at a density of 3 × 10^3^ cells/well in the medium as described in Section 2.4. After 24 h of culture, cells were treated with drugs as indicated in Figure 6 and Table 1 for 48 h. At harvest, cells were fixed by 10% trichloroacetic acid (TCA). After washing with distilled water, the viable cells were stained by SRB dye at 0.4% (w/v) in 1% acetic acid. The unbound dye was removed by repeated washing with 1% acetic acid and the plates were air-dried. The cell-bound SRB dye was subsequently solubilized with 10 mM trizma base, and the absorbance was read on a microplate reader at a wavelength of 570 nm. The absorbance is directly proportional to the cell number over a wide range.

### 2.7. Semiquantitative Reverse-Transcription Polymerase Chain Reaction (RT-PCR)

Total RNA was extracted separately from SP cells and non-SP cells using TRIzol reagent according to the manufacturer's instructions and was reverse-transcribed by using a First-Strand cDNA Synthesis Kit (Fermentas, Lithuania) as described in instructions. The RT-PCR was carried out in a 50 *μ*L reaction mixture that contained 1 *μ*g of cDNA as template, 1 *μ*M specific oligonucleotide primer pair, and 25 *μ*L Taq mixture containing 0.5 unit of Taq DNA polymerase. The PCR primers used for amplification were as follows: *ABCG2*, 5′-CTGAGATCCTGAGCCTTTGG-3′ and 5′-TGCCCATCACAACATCATCT-3′ for a 380 bp fragment; **β*-catenin*, 5′-ACTGGCAGCAACAGTCTTACC-3′ and 5′-TTTGAAGGCAGTCTGTCGTAAT-3′ for a 253 bp fragment; *CD133*, 5′-TCTCTATGTGGTACAGCCG-3′ and 5′-TGATCCGGGTTCTTACCTG-3′ for a 396-bp fragment; *SMO*, 5′-ATCTCCACAGGAGAGACTGGTTCGG-3′ and 5′-AAAGTGGGGCCTTGGGAACATG-3′ for a 369-bp fragment; *OCT-4,* 5′-GAC AACAATGAAAATCTTCAGGAGA-3′ and 5′-TTCTGGCGCCGGTTACAGAACCA-3′ for a 218 bp fragment; *Gli*, 5′-CAGAGAATGGAGCATCCTCC-3′ and 5′-TTCTGGCTCTTCCTGTAGCC-3′ for a 413 bp fragment; *GAPDH*, 5′-ACCACAGTCCATGCCATCAC-3′ and 5′-TCCACCACCCTGTTGCTGTA-3′ for a 249 bp fragment. The PCR products were separated by electrophoresis in 2% agarose gel.

### 2.8. Preparation of Cytoplasmic and Nuclear Proteins

Cytoplasmic and nuclear extracts of cells were prepared using the Nuclear Extraction Kit (Panomics). Briefly, harvested cells (1 × 10^6^ cells/6 cm plate) were washed twice with 5 mL cold 1 × PBS. A 0.5 mL aliquot of Buffer A working reagent (a combination of 0.5 mL 1 × Buffer A, 5 *μ*L DTT, 5 *μ*L protease inhibitor cocktail, and 20 *μ*L 10% IGEPAL) was added to each plate. The plate was transferred to an ice bucket on a rocking platform at 150 rpm for 10 min. Each sample was centrifuged at 14,000 × g for 3 min at 4°C. The supernatant (cytoplasmic fraction) was removed and the pellet kept on ice. A 75-mL aliquot of Buffer B working reagent (a combination of 0.5 mL 1 × Buffer B, 5 *μ*L DTT, and 5 *μ*L protease inhibitor cocktail) was added to each pellet and vortexed at the highest setting for 10 sec. Each sample was then placed in ice bucket and shook in rocking platform at 150 rpm for 2 h. After centrifugation at 14, 000×g for 5 min at 4°C, the supernatant (nuclear fraction) was transferred to a new Eppendorf tube for the measurement of the protein concentration of each sample, and was stored at –80°C.

### 2.9. Western Blotting

Samples (10 *μ*g) of cytoplasmic or nuclear proteins were size-fractionated electrophoretically by a 10% polyacrylamide SDS-PAGE gel and transferred onto a PVDF membrane using the Bio-Rad Mini-Protean electrotransfer system. The blots were subsequently incubated with 5% skim milk in PBST for 1 h to block nonspecific binding and were probed overnight at 4°C with the antibodies against total *β*-catenin (1 : 1000, Cell Signaling), Lamin (1 : 5000, Santa Cruz), and *β*-tubulin (1 : 5000, Santa Cruz). The membranes were sequentially detected with an appropriate peroxidase-conjugated secondary antibody incubation at room temperature for 1 h. Intensive PBS washing was performed after each incubation step. After the final PBS washing, signals were developed using the ECL (enhanced chemiluminescence) detection system and Kodak X-OMAT Blue Autoradiography Film.

### 2.10. Combination Index (CI) Measurements

Combination index (CI) between THL and doxorubicin (St. Louis, MO, USA) was obtained by a computer program (CalcuSyn) based on the median effect equation of *Chou and Talalay* [15]. The CI values below 1 indicate synergistic effects whereas those equal or close to 1 are additive and those above 1 are antagonistic. The analysis used in this study was under the assumption of mutual nonexclusiveness of the mechanism of drug action.

### 2.11. Tumor Xenografts on NOD/SCID Mice

The effects of THL on the tumorigenicity of Huh7 SP cells were evaluated on NOD/SCID mice. Huh7 SP cells (1 × 10^4^) were pretreated with or without 2 mg/mL of THL for 48 h, and all of the cells were then collected and injected subcutaneously into NOD/SCID mice. Forty days after inoculation, the final tumor size was measured with a caliper (calculated volume = shortest diameter^2^ × longest diameter/2). The animal study was approved by the NHRI Institutional Animal Care and Use Committee (Approved Protocol no. NHRI-IACUC-097051-A).

### 2.12. Statistical Analysis

The experiments were performed in triplicate, and the data represent means ± SD. Statistical significance (*P* < 0.05) was assessed by analysis of variance (ANOVA) followed by Student's *t*-test.

## 3. Results

### 3.1. Detection of Side Population in Human Hepatoma Cells

**Figure 1:**
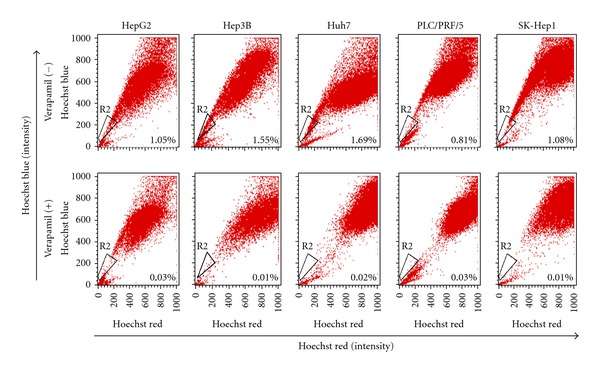
Existence of SP cells in the established hepatoma cell lines. Hepatoma cell lines were plated one day before analysis to achieve approximately 30% confluence on the day of analysis. The cells were detached, labeled with the Hoechst 33342 in the presence or absence of 50 *μ*M verapamil and then analyzed by flow cytometry. The SP cells which disappeared in the presence of verapamil (lower panel) are outlined and shown as a percentage of the total cell population.

**Figure 2:**
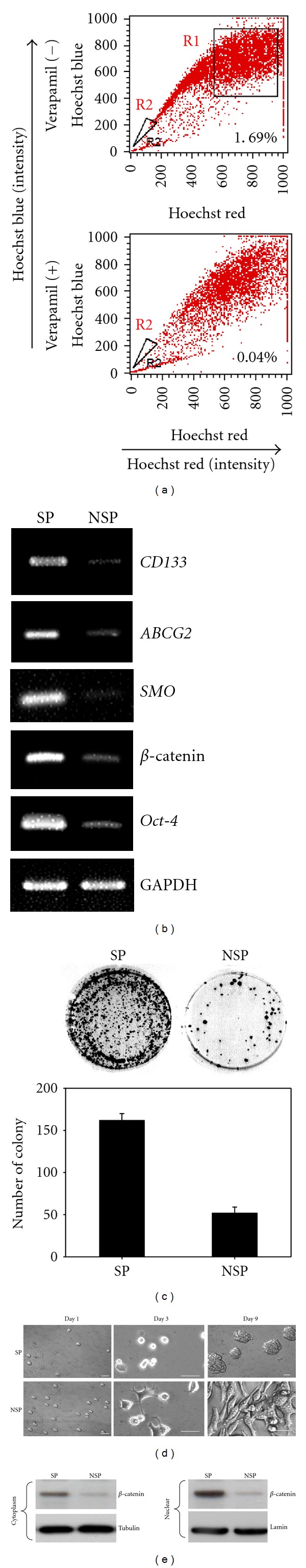
Huh 7 side population cells possessed higher clonogenicity and expression of stem cell markers. (a) Cell sorting of Huh7 cells using Hoechst 33342. The R2 gate represented the SP cells (1.69% of total cells) and the R1 gate represented the non-SP cells. Both of them were collected for the subsequent experiments. (b) RT-PCR assay showed that the SP cells possessed higher expression of stem cell markers. (c) Clonogenic assay showing the clonogenicity of SP cells was markedly higher than that of non-SP cells. (d) Cell morphology of freshly sorted SP and non-SP cells at days 0, 3, and 9 after seeding. As shown in the photographs, the differences in the shapes of SP (small and round in shape) and non-SP cells (fibroblast-like) were remarkable. (e) Protein expression and nuclear location of *β*-catenin in SP and non-SP cells. The SP cells had much higher nuclear *β*-catenin than the non-SP cells. Similar phenomenon was also observed in the cytoplasmic *β*-catenin level. Scale bar, 50 *μ*m. Similar results of clonogenicity and stemness genes expression were also obtained in HepG2 and Hep3B SP cells (data not shown). NSP: non-SP cells.

 To determine whether the selected hepatoma cell lines contained SP cells, we stained these cells with Hoechst 33342, which could be actively extruded by verapamil-sensitive ABC transporters. Representative results analysed by flow cytometry were shown in Figure 1. A small percentage of SP cells were found in 1.05% of HepG2, 1.55% of Hep3B, 1.69% of Huh7, 0.81% of PLC/PRC/5, and 1.08% of SK-Hep1 cells, respectively, which were decreased markedly in the presence of verapamil. When preincubated with verapamil for 90 min, the percentage of side population cells shown on the flow cytometer dropped to 0.04% of the total cells (Figure 2(a)). This result is consistent with the reports that Hoechst 33342 exclusion is verapamil sensitive. The SP cells were then collected for the subsequent experiments.

### 3.2. Side Population Cells Have Distinct Stem Cell Properties

As shown in Figure 2(a), the R2 gate showed lower Hoechst 33342 intensity indicated the SP cells, and the R1 gate showed higher Hoechst 33342 intensity indicated the non-SP cells. Like normal stem cells, the RT-PCR analysis reveals that Huh7 SP cells expressed higher levels of *ABCG2, CD133, SMO, *β*-catenin,* and *Oct4* mRNA than non-SP cells, suggesting that the SP cells have, at least a part, distinct intrinsic properties of stem cells (Figure 2(b)). After 9 days of culture, most colonies had formed and the number of colonies in SP and non-SP cells was 165 and 55, respectively (Figure 2(c)). The spheroid morphology of SP cells was markedly distinct from the fibroblast-like shape of non-SP cells (Figure 2(d)). In addition, both the nuclear and cytoplasmic *β*-catenin protein levels of SP cells were markedly higher than those of non-SP cells. The difference between the nuclear *β*-catenin levels in SP and non-SP cells was even much higher than that between the cytoplasmic levels (Figure 2(e)). This phenomenon was consistent with that shown in Figure 2(b) and reflected the cancer stemness of Huh7 SP cells.

### 3.3. THL Decreased Proportion of SP Cells in Human Hepatoma Cell Lines

**Figure 3:**
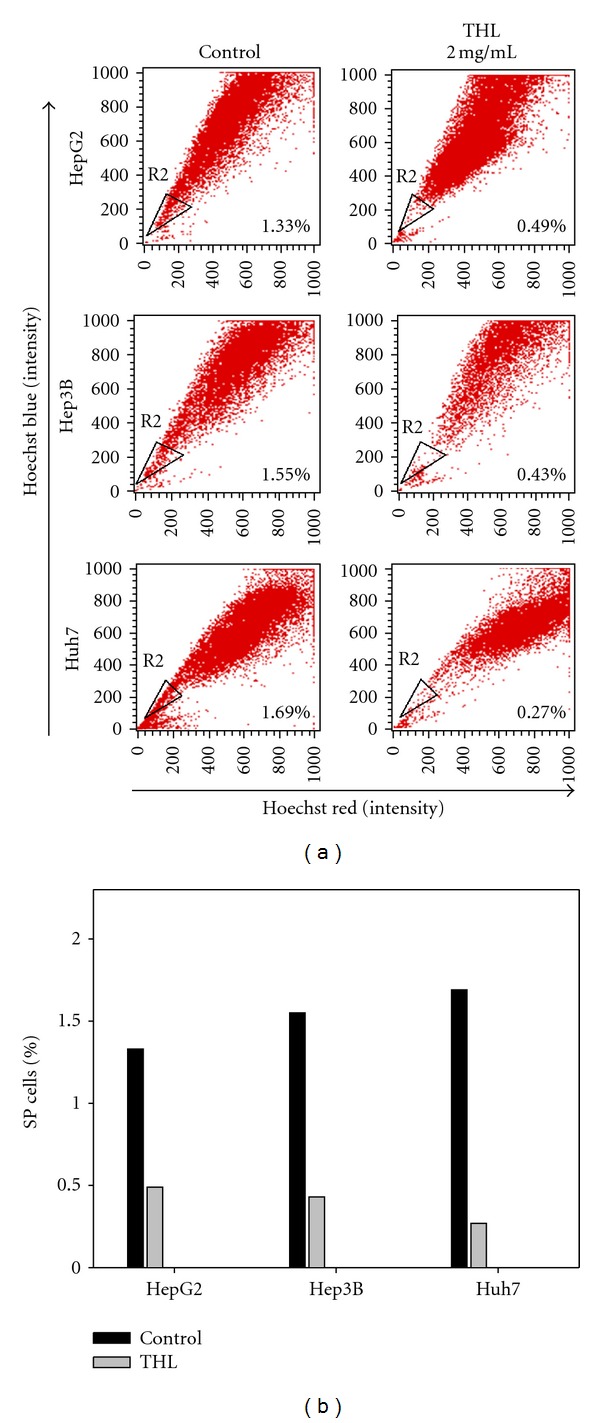
THL decreased the proportion of SP cells in hepatoma cell lines. (a) Hepatoma cells were incubated with 2 mg/mL of THL for 2 days, and then the proportion of SP was gated and analyzed after incubation with Hoechst 33342. Representative data were shown. (b) The data of Figure 3(a) presented in bar graph format.

To evaluate the effects of THL targeting on hepatoma CSCs, we analyzed its inhibitory effects on side population by using flow cytometry and Hoechst 33342 efflux assays. After 2 days of THL treatment at dose of 2 mg/mL, the proportions of SP cells were reduced from 1.33% to 0.49% in HepG2, 1.55% to 0.43% in Hep3B, and 1.69% to 0.27% in Huh7 cells, respectively, as shown in Figure 3.

### 3.4. THL Suppressed Growth and Colony Formation of Huh7 SP Cells

**Figure 4:**
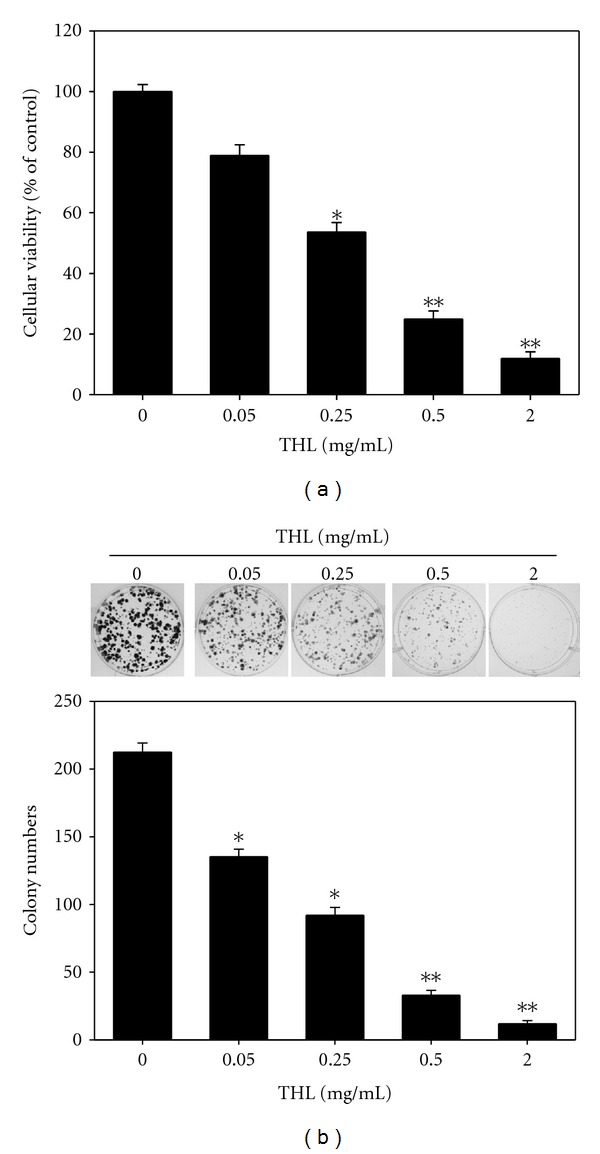
THL inhibited the proliferation and colony formation of Huh7 SP cells. (a) Proliferation inhibition effects of different doses of THL on Huh7 SP cells. (b) Colony formation inhibition effects of THL on Huh7 SP cells. Huh7 SP cells were seeded into 6-well plates in DMEM/F12 medium (supplement with B27 supplement, bFGF and EGF). After 24 h, the cells were treated with the indicated concentrations of THL for 14 days. At harvest, the SP cells were fixed and stained with crystal violet to visualize the colonies for counting. *P < 0.05; **P < 0.01, indicating statistical significance as compared to the untreated control SP cells.

To further investigate how effective was THL against hepatoma SP cells, the growth and colony formation were measured. As expected, THL dose-dependently inhibited both the proliferation and colony formation of Huh7 SP cells. As shown in Figures 4(a) and 4(b), the cell viability and colony number were significantly reduced from 100 ± 2.3% to 11.9 ± 2.1 % and 200 ± 5.3 to 21.3 ± 2.3, respectively, by THL at dose of 2 mg/mL.

### 3.5. Downregulation of Cancer Stemness Genes by THL

**Figure 5:**
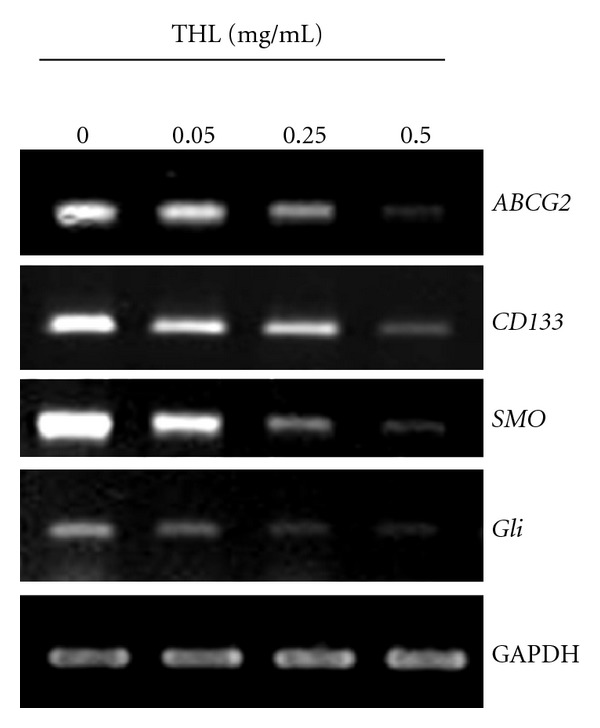
THL dose-dependently diminished the mRNA expression of *ABCG2, CD133, SMO,* and *Gli* in Huh7 SP cells. Huh7 SP cells were treated with varying concentrations of THL for 48 h, and then the mRNA levels were analyzed by RT-PCR analysis. The reference *GAPDH* was used as the loading control.

To determine the mechanisms underlying the effects of THL on the elimination of Huh7 SP cells, the expression of several stemness genes that were responsible for stem cell self-renewal, proliferative capacity, or lineage differentiation was examined by RT-PCR. As shown in Figure 5, the mRNA levels of *ABCG2* and *CD133* were decreased in a dose-dependent manner after 2 days of THL treatment. Moreover, the Hedgehog signaling pathway genes such as *SMO* and its downstream *Gli* were also significantly downregulated by THL. These results suggested the mechanisms responsible for the eradication of Huh7 SP cells by THL are probably through multiple molecular targeting effects.

### 3.6. The Synergistic Inhibitory Effect of THL and Doxorubicin in SP Cells

**Figure 6:**
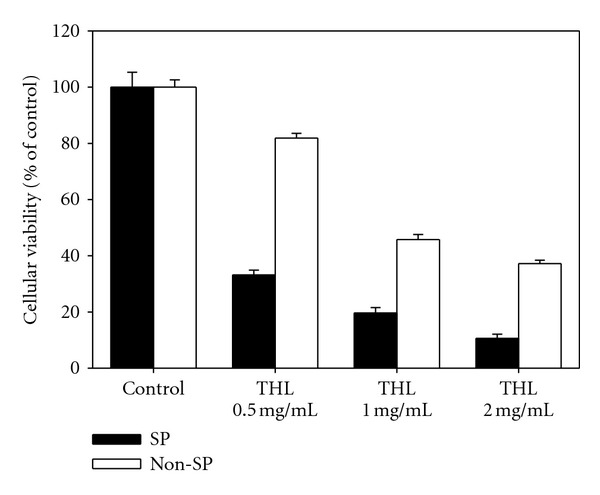
The growth inhibition effects of THL on Huh7 SP and non-SP cells. Both the SP and non-SP cells were treated with varying concentrations of THL for 48 h and then the cell viabilities were determined by SRB assay. The SP cells appeared to be more sensitive to THL than the non-SP cells.

To further investigate the CSC targeting effects of THL, we compared the effects of THL on the growth inhibition of Huh7 SP and non-SP cells. The result showed that THL appeared to preferentially inhibit the proliferation of SP cells (Figure 6). Next, we studied whether the effect of doxorubicin against Huh7 SP cells could be synergized by combining with THL. By calculation, THL (65 *μ*g/mL) or doxorubicin (250 nM) alone produced only 36% and 5% decrease in the viability of Huh7 SP cells as compared to control, respectively. However, simultaneous treatment with these two drugs resulted in a 63.6% decrease in the viability as shown in Table 1. In addition, the combined index (CI) values of this combination (using mutually nonexclusive model) were all well below 1, indicating the synergistic combination effects of doxorubicin with THL. 

### 3.7. THL Decreased the Number of Sphere Formed by Huh7 SP Cells and Suppressed Their Tumorigenicity in NOD/SCID Mice

**Figure 7:**
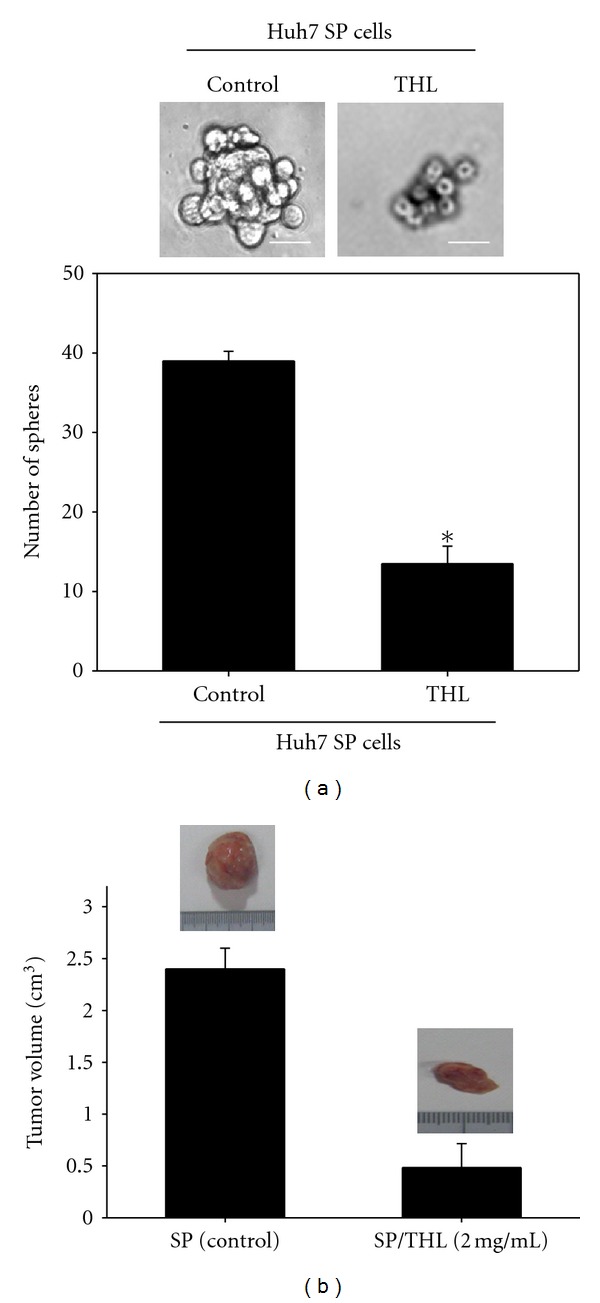
THL decreased the number of sphere and suppressed the tumorigenicity of Huh7 SP cells. (a) The size and number of spheres formed by Huh7 SP cells were decreased after treatment with THL. Representative photos (upper) and the number of Huh7 SP spheres (lower) after treatment for 48 h with vehicle and THL, respectively. Scale bar, 50 *μ*m. (b) The tumorigenicity of Huh7 SP cells was significantly suppressed by pretreatment with 2 mg/mL of THL for 48 h. The photographs of representative tumor mass in each group were shown. On day 0, untreated and THL-pretreated Huh7 SP cells were subcutaneously injected into the NOD/SCID mice. The animals were sacrificed and the sizes of tumor were determined at day 40 after inoculation of Huh7 SP cells. The untreated Huh7 SP cells formed tumor in 5 out of 5 mice, while the THL-treated SP cells formed tumor only in 2 out of 5 mice. *P < 0.05, indicating statistical significance as compared to the untreated control SP cells.

The cancer stem cell targeting effects of THL were also evaluated on the tumor sphere formation and tumorigenicity of Huh7 SP cells, which formed tumors in 5 out of 5 NOD/SCID mice by 10^4^ cells injected while the parental Huh7 cells formed tumors in 5 out of 5 mice by 10^7^ cells injected and the non-SP cells could not form any tumor even by 10^7^ cells injected (data not shown). As shown in Figure 7(a), at dose of 2 mg/mL, the number of tumor spheres was reduced from 39 ± 1.2 of control to 13.5 ± 2.2 by THL, indicating its inhibitory effects on the self-renewal of Huh7 SP cells. In the xenograft NOD/SCID mice model, the tumorigenicity of THL-pretreated Huh7 SP cells was significantly reduced compared with the untreated SP cells (Figure 7(b)). The untreated Huh7 SP cells formed tumor in 5 out of 5 mice, while the THL-treated SP cells formed tumor only in 2 out of 5 mice at the time of 40 days after SP cells inoculation. In addition, the average final tumor size was reduced from 2.4 ± 0.2 cm^3^ to 0.48 ± 0.2 cm^3^, suggesting the inhibitory effect of THL on the tumorigenicity of Huh7 SP cells.

## 4. Discussion

The recent discovery of cancer stem cells has great importance both for development of new strategy to combat malignant tumor and evaluation of pitfalls and benefits of current therapeutics. Since the SP isolated from hepatoma cells had been reported to possess high proliferation potential, tumorigenicity, and antiapoptotic properties compared with those of non-SP cells [16, 17], we used the SP analysis as a tool to evaluate the effects of THL on elimination of cancer stem cells (CSCs). Besides the method of SP analysis, many researches detected and isolated the CSCs by tracking the specific CSCs surface CD markers that were not expressed on the bulk of the other cancer cells [18]. In addition to the multidrug-resistant efflux pump gene *ABCG2*, which was responsible for effluxing Hoechst 33342, the sorted SP cells also expressed higher levels of not only *CD133*, a putative cell surface marker for hepatoma stem cells isolation [19], but also other stemness genes such as *SMO*, *β-catenin Oct-4* as well as clonogenicity than those in non-SP cells. The distinct protein expression and nuclear location of *β*-catenin in SP cells further reflected their cancer stemness. These results indicated the rationality of using SP cells to evaluate the activities of potential targeting agents on CSCs. Nevertheless, Wu et al. [20] had examined mesenchymal tumors ranging from benign to high-grade sarcomas and found that high aggressive tumors were prone to possess highly proportion of SP cells, which might correlate with the poor prognosis in mesenchymal tumors. In accordance with our point, Wu's study also supported that the SP cells were an important therapeutic target for drug intervention.

Unlike the ABCG2 inhibitor verapamil, THL decreased not only the proportion of SP cells, but also the cell viability and colony formation of SP cells. In addition, THL dose-dependently inhibited the expression of important stemness genes such as the putative hepatoma stem cell marker *CD133* [19], as well as the Hedgehog signaling pathways components *SMO* and *Gli* [21]. CD133/prominin-1, a pentaspan membrane glycoprotein, is an important cancer stem cell surface marker in various solid tumors, including liver cancer [22]. It had been shown that the CD133(+) Huh7 hepatoma cells performed a higher proliferative potential, and tumorigenicity, and lower mRNA expressions of mature hepatocyte markers than the CD133(−) population [19]. The suppressing effects of THL on the *CD133* of Huh7 SP cells indicated its potential in targeting CSCs in hepatoma. Hedgehog signaling, a crucial factor in regulating self-renewal of stem cells, is frequently aberrantly activated in CSCs [23] and thus became a potential target for cancer therapy [23, 24]. Recent studies also showed that activation of Hedgehog signaling is critically related to CSCs and EMT (epithelial-mesenchymal transition) features in many types of cancers including colon, gastric, esophagus, liver, and other cancers [25]. The diminished expression of *SMO* and its downstream *Gli* by THL further revealed its potential role on the eradication of hepatoma CSCs.

The ABCG2, which highly expressed in hepatoma SP cells, is associated with drug efflux related to the resistance of doxorubicin [26] and considered as a cause of poor response of hepatoma patients to this drug. Accordingly, our results showed that THL diminished *ABCG2* expression in SP cells and synergized the effects of doxorubicin against Huh7 SP cells. On the other hand, CD133 was shown to confer chemoresistance by activation of the AKT/PKB and Bcl-2 cell survival response in hepatoma cells [27], and suppression of the Hedgehog pathway could also sensitize the hepatoma cells to chemotherapeutic agent [28]. Regarding the profound effects of THL on these two important targets (Figure 5), the synergistic combination effects might also attribute to the THL-suppressed CD133 and Hedgehog signaling pathway.

Like THL, medicinal plants or phytochemicals are potential sources for therapeutics targeting on CSCs. For example, berberine and cannabinoids had been reported to diminish the cancer stem-like cells in breast and brain cancer, respectively [8, 9]. Various agents that directly modulate CSCs had been evaluated *in vivo* and *in vitro* [29]. However, the biology of CSCs is extremely complex and accompanied with a considerable crosstalk and redundancy in signaling pathways. Hence, targeting only single molecule or pathway inside CSCs may exert limited benefit for treatment. The combination of CSCs eliminating compounds was thus proposed to enhance the efficacy. Combination of curcumin and piperine was found to further reduce the proportion of breast CSCs than when either drug was used alone [10]. Cotreatment with sulforaphane, a broccoli isothiocyanate, could enhance the sorafenib-mediated elimination of pancreatic CSCs *in vitro* and synergize its effects on tumor size reduction *in vivo* [30]. As THL consists mainly of extracts from 14 Chinese herbs and possessing activities of multiple oncogenic signaling pathways inhibition [12], its significant effects against Huh7 SP cells may be resulted from the synergistic combination effects of each active components contained in this herbal mixture.

A recent proposed mechanism to eradicate CSCs is epigenetic modulation by depleting the DNA methyltransferase 1 (DNMT1), which plays a critical role on the aberrant hypermethylation of DNA. DNMT1 depleting agents such as Decitabine and its analog Azacitidine had been shown to inhibit cultured solid tumor stem-like cells and diminish tumorigenicity [31]. Our previous study had showed that THL could intensively decrease the protein level of DNA methyltransferases 1 (DNMT1), an important enzyme for the aberrantly DNA methylation, in acute promyelocytic leukemia cells [12]. In addition, we also found that the expressions of DNMTs (DNMT1, 3a, 3b) were much higher in Huh7 SP cells compared with those of non-SP cells (data not shown). Therefore, THL might also affect the DNMT1 of Huh7 SP cells, which resulted in its eradication effects on these cells. Hence, the involvement of epigenetic modulation through DNMT1 depletion on THL-induced elimination of Huh7 SP cells is worth of further investigation.

Taken together, searching and identifying the potential cancer stem cell targeting agents, either from nature products or synthetic chemical compounds, are believed to provide substantial benefit for curing cancer. As combination effect may be more efficient for CSCs elimination, the traditional herbs or recipes could be considered as a potential source for CSCs targeting therapeutics. Based on the results shown in this study, future clinical trial to evaluate the complementary effects of THL on the relapse free survival of hepatoma patients after surgery is warranted.

## 5. Conclusions

We investigated the effects of Chinese herbal mixture *Tien-Hsien Liquid* (THL) on cancer stem-like side population (SP) in hepatoma Huh7 cells. THL significantly reduced the viability, clonogenicity, and tumorigenicity of Huh7 SP cells. The underlying mechanisms of SP elimination were related to the inhibition on the expression of stemness genes such as *ABCG2*, *CD133*, *SMO,* and *Gli*. Moreover, combination with THL could synergize the effect of doxorubicin against Huh7 SP cells. Our data indicate that THL may act as a cancer stem cell targeting therapeutics and be regarded as complementary and integrative medicine for the treatment of hepatoma.

## Figures and Tables

**Table 1:** Synergistic combination effects of THL and doxorubicin against Huh7 SP cells (using mutually nonexclusive model). At fixed dose of THL and various doses of doxorubicin, the CI values were all well below 1, indicating the synergistic combination effects. Inhibition (fraction affected) values ranged from 0 (no inhibition) to 1 (100% inhibition). The higher the dose of doxorubicin used, the more proportion of cell viability was inhibited.

Mutually non-exclusive CI for experimental values			
THL (*μ*g/mL)	Dox (nM)	Fraction affected	CI
65	250	0.636	0.582
65	500	0.665	0.656
65	1000	0.83	0.512
65	2000	0.91	0.455
